# MTA2 triggered R-loop trans-regulates BDH1-mediated β-hydroxybutyrylation and potentiates propagation of hepatocellular carcinoma stem cells

**DOI:** 10.1038/s41392-021-00464-z

**Published:** 2021-04-02

**Authors:** Heng Zhang, Zhi Chang, Lu-ning Qin, Bin Liang, Jing-xia Han, Kai-liang Qiao, Cheng Yang, Yan-rong Liu, Hong-gang Zhou, Tao Sun

**Affiliations:** 1 State Key Laboratory of Medicinal Chemical Biology and College of Pharmacy, Nankai University, Tianjin, China; 2 Tianjin Key Laboratory of Early Druggability Evaluation of Innovative Drugs and Tianjin Key Laboratory of Molecular Drug Research, Tianjin International Joint Academy of Biomedicine, Tianjin, China; 3 Molecular Pathology Institute of Gastrointestinal Tumors, Affiliated Hospital of Jining Medical University, Jining Medical University, Jining, Shandong China; 4 Department of Gastroenterology and Hepatology, Tianjin Medical University General Hospital, Tianjin Institute of Digestive Disease, Tianjin, China

**Keywords:** Cancer stem cells, Cell biology, Gastrointestinal cancer

**Dear editor,**

MTA2 is associated with invasively malignant phenotypes in many types of cancers,^1^ but the molecular mechanism remains unclear. Studies on the role of MTA2 mostly concentrated on it as a component of the NuRD complex, which functions via the NuRD complex to inhibit the transcription of downstream target genes.^2^ Whether MTA2 could directly exert transcriptional regulation independent of the transcription factor remains unclear and is the focus of the present study.

The immunohistochemical staining image of MTA2 provided by the Human Protein Atlas revealed that MTA2 was highly expressed in hepatocellular carcinoma (HCC) cells and localized in the nuclei. The analysis results based on The Cancer Genome Atlas (TCGA, see supplementary Table S1 for patient information) showed that MTA2 mRNA in HCC was remarkably upregulated (Fig. 1a). The high expression level of MTA2 was related to short overall survival (Fig. 1b) and disease-free survival. Thus, MTA2 is a marker of the malignant progression of HCC. MTA2 was highly expressed in cases with high α-fetoprotein (AFP) levels in peripheral blood. Therefore, MTA2 may be related to HCC malignance (Supplementary Fig. S1).

**Fig. 1:**
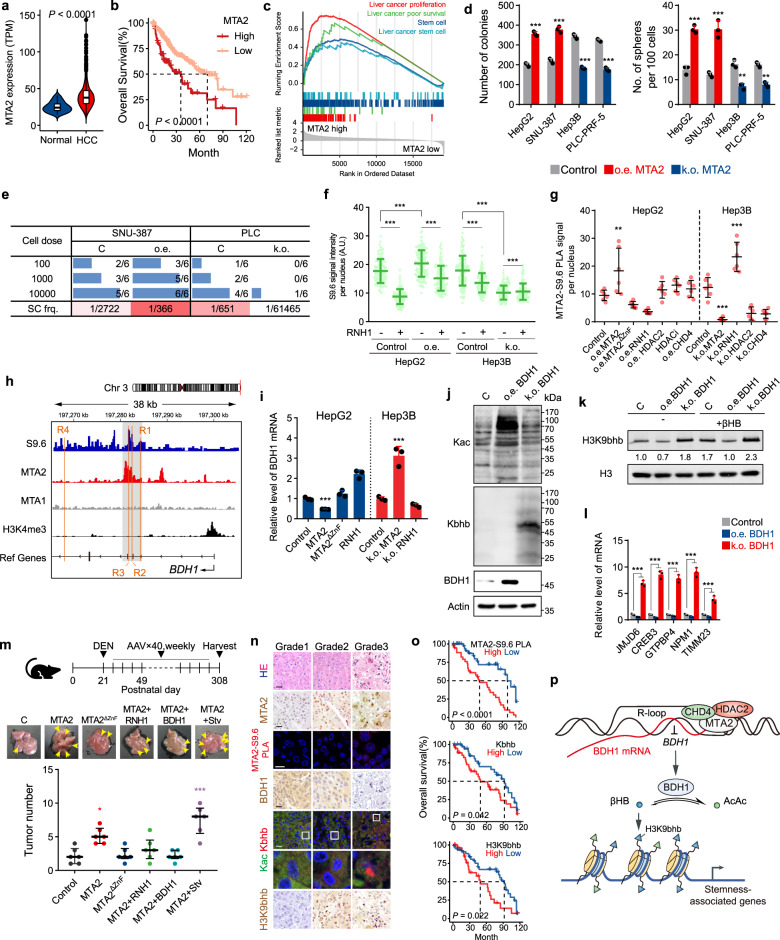
MTA2 triggered R-loop and potentiated propagation of hepatocellular carcinoma stem cells. **a** MTA2 mRNA expression level (TPM) in adjacent tissues (*n* = 50) and HCC tissues (*n* = 371) in the TCGA dataset. **b** Kaplan–Meier curves showing percentage of the overall survival of the high and low expression of MTA2. (*n* = 371). **c** GSEA analysis of patients with high or low MTA2 expression. **d** Plate colony formation number (left) and oncosphere formation number (right) obtained after overexpressing or knocking out MTA2 in four HCC cell lines. *n* = 3, biological replicates. **e** Limiting dilution assay was performed to compare the frequency of stem cells (SC) in control cells, overexpression or knockout of MTA2 in two HCC cell lines. *n* = 6, biological replicates. **f** Quantification of S9.6 nuclear signal after overexpression or knockout of MTA2 in two HCC cell lines. The nucleus was co-stained with nucleolin antibody and treated with RNase H1 as indicated. A.U., arbitrary units. In each group 100 cells were counted. **g** MTA2 and S9.6 antibodies were used for PLA to perform confocal imaging (**h**) and quantification (**i**) of MTA2-induced R-loop in two HCC cell lines. HADCi: suberoylanilide hydroxamic acid (SAHA), an HDAC inhibitor. In each group 6 cells were counted. (**h**) Peak plot of S9.6 DRIP, MTA2, MTA1, and H3K4me3 ChIP at the BDH1 gene region. **i** qRT-PCR of BDH1 mRNA after indicated treatments in two HCC cell lines. *n* = 3, biological replicates. **j** Western blot of Kac and Kbhb after overexpression or knockout of BDH1 in PLC-PRF-5 cells. **k** Western blot of H3K9bhb on the condition that the precursor βHB were supplemented. **l** qRT-PCR of five genes filtered in (**e**) after indicated treatments in PLC-PRF-5 cells. *n* = 3, biological replicates. **m** Representative images of livers and quantification of tumor numbers from 11-month-old DEN-treated mice after indicated treatments. Stv: starvation treatment. *n* = 6, biological replicates. **n** HE staining, MTA2 IHC, MTA2-S9.6 PLA, BDH1 IHC, Kac/Kbhb IF, H3K9bhb IHC, and CD133 IHC of the same tissue. Scale bar: 20 μm. **o** KM survival analysis of high and low groups according to MTA2-S9.6 PLA, Kbhb, and H3K9bhb signal. (*n* = 75). **p** Proposed model for the MTA2-Rloop-BDH1-Kbhb axis in HCC stemness. *p < 0.05, **p < 0.01, ***p < 0.001, Student’s *t*-test

To investigate the biological functions of overexpressed MTA2 in HCC, we proportionally divided the cases in the TCGA into two groups based on MTA2 expression level (50% cut-off) for gene set enrichment analysis (GSEA). The results showed that MTA2 enhanced the upregulation of HCC stemness. (Fig. 1c and Supplementary Fig. S1I) We also found that MTA2 expression level was positively correlated with cancer stem cell markers CD44, PROM1 (CD133), and POU5F1 (Oct4). Considering that SOX9 is an important stem cell marker of hepatocytes and HCC, we also confirmed that MTA2 was positively correlated with the expression of SOX9 by immunohistochemistry on 340 cases with HCC. (Supplementary Fig. S1) The plate clone formation assay and tumorsphere formation assay (Fig. 1d, Supplementary Fig. S1O and S1P) showed that HCC cells with overexpressed MTA2 enhanced HCC stemness. HCC cells with overexpressed MTA2 upregulated the expression levels of HCC stem cell markers CD44, CD133, SOX9, and EpCam and increased the proportion of the CD133-positive subset (Supplementary Fig. S1). The in vivo tumor-forming rate of the group with overexpressed MTA2 significantly improved. However, the knockout of MTA2 obtained opposite results (Fig. 1e).

We further investigated the molecular mechanism of MTA2 in promoting HCC stemness. Given that MTA2 is a component of the NuRD complex, the enhancement of stemness by MTA2 may be related to the transcriptional inhibition of the NuRD complex. Through bioinformation analysis and biochemistry assay, we found a complex, namely, MTA2-HDAC2-CHD4, formed by a part of the NuRD complex in CD133^+^ HCC cells. The components of NuRD complex was different in the CD133^+^ and CD133^−^ HCC cells, and its function was inhibited in the former. Hence, the MTA2-HDAC2-CHD4 complex is closely related to HCC stemness. (Supplementary Fig. S2) We performed chromatin immunoprecipitation (ChIP) assay on MTA2 in the two groups of cells to determine how the complex functions and what target genes are regulated. In the CD133^+^ group, we found a large amount of RNA mixed in the DNA fragments acquired by MTA2 antibody ChIP. After treatment with a variety of ribonucleases (RNases), the RNA content was sensitive to RNaseH, which could degrade the RNA strand in RNA-DNA hybrids. RNA that occurs in the transcriptional process can pair with the template DNA to form an R-loop in several cases, thereby pausing transcription.^3^ Immunofluorescence staining was performed on the two groups by using monoclonal antibody S9.6 that can recognize DNA-RNA hybrids. The results indicated that the R-loop level in CD133^+^ cells was high (Supplementary Fig. S3). We analyzed the published HCC single-cell sequencing data GSE103866^4^ and found that although the R-loop increased in HCC stem cells, the level of RNaseH did not increase. The immunofluorescent staining using S9.6 antibody showed that the overexpressed MTA2 increased the R-loop, whereas MTA2 knockout reduced the R-loop (Fig. 1f and Supplementary Fig. S3G). ZnF domains were required for MTA2 to induce R-loop formation. The MTA2-induced R-loop was observed by PLA, and was positively correlated with MTA2 expression (Fig. 1g and Supplementary Fig. S3K). The MTA2-induced R-loop neither changed significantly with overexpressed MTA2^ΔZnF^, HDAC2, or CHD4 nor was it upregulated by adding the HDAC inhibitor. These results proved that MTA2 recruited HDAC2/CHD4 to form a new complex and triggered R-loop.

The target genes regulated by MTA2-triggered R-loop were investigated. On the basis of the peak of S9.6 DRIP-seq and MTA2 ChIP-seq, we found that BDH1 was the unique gene that co-expressed with MTA2 and CD133 and has a significant effect on the overall survival of HCC. The expression of BDH1 was negatively correlated with MTA2 and CD133 (Supplementary Figs. S4A, S4B and Table S3). In the BDH1 open reading frame, S9.6 and MTA2 had strong signal peaks at the same position (hg19: 197, 281, 756-197, 282, 861, 1105 bp, Fig. 1h), indicating that the MTA2-related R-loop appeared at this position. We established a dual-luciferase reporter assay to measure BDH1 transcription activity, and the signal was negatively associated with MTA2. Furthermore, the level of BDH1 mRNA (Fig. 1i) and protein was negatively associated with MTA2. RNaseH could rescue the effects of MTA2, and MTA2^ΔZnF^ had no significant influence (Supplementary Fig. S4). BDH1, as a major rate-limiting enzyme in the metabolic process of ketone bodies, controls the transformation between acetoacetic acid (AcAc) and βHB. Given that the metabolites of AcAc and βHB are the precursors of acylation precursors, the effects of BDH1 on Kac and Kbhb were examined. The Western blot (Fig. 1j) and FCM results revealed that BDH1 could upregulate the Kac level and downregulate that of Kbhb (Supplementary Fig. S5). H3K9bhb is the most enriched at the TSSs of active genes among Kbhb marks.^5^ H3K9bhb level was negatively correlated with BDH1 (Fig. 1k). Furthermore, five target genes, namely, JMJD6, GREB3, GTPBP4, NPM1, and TIMM23, which led to poor prognosis in HCC, were also verified by TCGA dataset. The mRNA level (Fig. 1l) of the five genes was negatively correlated with BDH1. βHB and MTA2 increased the expression levels of the H3K9bhb and mRNA level of these genes, whereas BDH1 and RNaseH restored the effects of MTA2 (Supplementary Fig. S5). These results demonstrated that these genes could be regulated by the MTA2-Rloop-BDH1-Kbhb axis.

The effect of the MTA2-Rloop-BDH1-Kbhb axis on HCC stemness was verified by the plate clone formation assay and tumorsphere formation assay (Supplementary Fig. S6). DEN-induced HCC model showed that MTA2 increased the number of HCC tumors, and RNH1 and BDH1 rescued the number of HCC tumors increased by MTA2 (Fig. 1m). Immunohistochemistry or immunofluorescent staining was performed on MTA2, MTA2-induced R-loop, BDH1, Kac, Kbhb, H3K9bhb, and CD133 (Fig. 1n) by using the paraffin specimens from 340 cases with HCC (Supplementary Table S5). As shown in Fig. 1o, the high levels of the three indicators indicated poor prognosis (Supplementary Fig. S7).

We revealed that MTA2 could interact with HDAC2/CHD4, and transcriptionally inhibit BDH1 by R-loops, leading to the accumulation of βHB, the increase in H3K9bhb, and a waterfall effect on HCC formation and progression. The abnormal metabolism and microenvironment of the organism are also important conditions for tumor formation and progression (Fig. 1p).

## Supplementary information

Supplemental methods and figures

Table S1

Table S2

Table S3

Table S4

Table S5

## Data Availability

All data relevant to this work are included in this paper and Supplementary Information.
