# Supplementary material for: MTA2 triggered R-loop trans-regulates BDH1-mediated β-hydroxybutyrylation and potentiates propagation of hepatocellular carcinoma stem cells
Source: Signal Transduct Target Ther. 2021 Apr 2;6:135. doi: 10.1038/s41392-021-00464-z (PMC8016859; doi:10.1038/s41392-021-00464-z)
Supplement: Supplementary file 1 — Supplemental methods and figures [file 41392_2021_464_MOESM1_ESM.pdf]

Supplementary Materials for

MTA2 triggered R-loop trans-regulates BDH1-mediated β-Hydroxybutyrylation and potentiates propagation of hepatocellular carcinoma stem cells

Heng Zhang[1,2], Zhi Chang[1,2], Lu-ning Qin[1], Bin Liang[2], Jing-xia Han[2], Kai-liang Qiao[1], Hong-gang Zhou[1,*], Yan-rong Liu[2,4,*], Cheng Yang[1,2,*] and Tao Sun[1,2,3,5,*]

Correspondence to: tao.sun@nankai.edu.cn, cheng.yang@nankai.edu.cn, liuyanrong1984@163.com, honggang.zhou@nankai.edu.cn

This PDF file includes:

Materials and Methods
Supplementary Text
Figures. S1 to S12

Other Supplementary Materials for this manuscript include the following:

Table S1 to S5

Table S1. Patient information in TCGA LIHC dataset

Table S2. The original data of co-expression analysis of MTA2 with the other components of the NuRD complex and target genes of the NuRD complex in whole cases, CD133^{-} cases, and CD133^{+} cases in TCGA

Table S3. Co-expression and survival analysis of 96 genes whose open reading frame had MTA2 and S9.6 signals

Table S4. The original data of co-expression analysis of BDH1 with H3K9bhb-regulated genes in whole cases, $CD133^-$ cases, and $CD133^+$ cases in TCGA

Table S5. Patient information for IHC, PLA or IF and other analysis

MATERIALS AND METHODS

Cell culture

The human HCC cell lines Hep3B and SUN-387 were obtained from the Guangzhou CELLCOOK, PLC-PRF-5 and HepG2 were purchased from Nanjing Keygen Biotech. SK-Hep1 and SUN-387 cells were cultured in RPMI-1640 medium (Keygen Biotech), and HepG2 and PLC-PRF-5 cells were cultured in Dulbecco's modified Eagle's medium (Keygen Biotech) supplemented with 10% (v/v) fetal bovine serum (Thermo Fisher Scientific, USA) at 37 °C in humidified atmosphere containing 5% CO_2. The cell lines were tested to determine the presence of *Mycoplasma* before use. Complete cell identification was provided by Cellcook or KeyGen Biotech.

Overexpression and CRISPR knockout

HepG2, SNU387, Hep3B and PLC-PRF-5 cells were transfected using Lipo8000 (Beyotime) according to the manufacturer's instructions with MTA2, BDH1, RNH1, HDAC1, HDAC2, CHD3 and CHD4 overexpression plasmid (Sino Biological) or CRISPR Knockout Plasmids containing Cas9 and guide RNAs (Santa Cruz). Transfected cells were incubated for 48 h before puromycin selection. MTA2, BDH1, RNH1, HDAC1, HDAC2, CHD3 and CHD4 knockout was confirmed by RT-qPCR and western blotting. (Figure S8)

Quantitative PCR

Quantitative PCR was performed as described previously.[1] The sequences of the primers used are provided as follow.

Gene	Forward	Reverse
MTA2	CTCCTCGTCTCCCGGTTC	GTAAAACGACGGCCAGT
CHD3	CCGTCAGCATTGGGTGTGAA	TCTTGCGTTTTCGGGGTTTTC
HDAC1	CTACTACGACGGGGATGTTGG	GAGTCATGCGGATTCGGTGAG
HDAC2	ATGGCGTACAGTCAAGGAGG	TGCGGATTCTATGAGGCTTCA
CHD4	TGAGGGCAGCGACTATACTCC	GAGCAGATGATTTAGGCTCCTTT
RNH1	GAGCTGAGCGACGCTAGATG	GGGTTGACTCGAAGTGCAGAG
BDH1 (mRNA)	GTAAAACGACGGCCAGTATGCAGCCCAGTT TGACTTTC	CTCATCACCGCCTACACTGTC
BDH1(R1)	CCAGCTTTGCTGTGACAACC	ATGTGTCAGAGTGGACAGCG
BDH1(R2)	CATCCTACCCTTACCCCGCT	GCCCTCTACTCGGTTTGGTT
BDH1(R3)	CGTAAGCAGAGCGCGAGAA	CTCAACCAATGGGAAGCGTG
BDH1(R4)	GAGCACTGCTGGACTTCTCG	AGCGACTGTAAGAGCTGCAAA
CDH13	GTAAAACGACGGCCAGTCAGCCTCTACCCA ATGCTTTC	GCTCGGAATGACCTCCCTAC
SYT1	GGTTGGCTGTTTCCCAGTAAAAC	TTTTAAGAAGTACGGACCATCGG
TLE1	TGTCTCCCAGCTCGACTGTCT	AAGTACTGGCTTCCCCTCCC
JMJD6	CCCAGGACGACTGTGTCAG	GCCTCCACAAGTGTCCCTAA
CREB3	GTCTCCTTCTGCCTCCTCCTT	GTCTGAGCCGTCCAACCACT

GTPBP4	GTAAAACGACGGCCAGTGGAAGTGACGTACCTCAACCG	GGGAAATGATCCCGAGTACG
NPM1	GCGAGGTAGAAAGGAGTGGG	GACGGAATCACACCAGGGAA
TIMM23	GTAAAACGACGGCCAGTCATGTAACAATCAGGAGCTGGA	CTGGTTTCAGAGAGATGCAGG
GAPDH	CCAAGGTCATCCATGACAAC	TGTCATACCAGGAAATGAGC

Western blot analysis

Cells were washed with PBS and lysed in ice-cold lysis buffer with protease Inhibitor Cocktail (Sigma) on ice for 30 min. Lysates were separated through electrophoresis and transferred onto polyvinylidene difluoride membranes (Millipore). The membranes were blocked, incubated with primary antibody against MTA2 (Bioss), SOX9 (Santa Cruz), CD44, CD133, MTA1, MTA3, HDAC1, HDAC2, CHD4, BDH1, H3 (1:1,000, Affinity), Kac, Kbhb, and H3K9bhb (1:1,000, PTM Biolabs) at 4 °C overnight, and incubated with a horseradish peroxidase-conjugated goat anti-rabbit or goat anti-mouse IgG secondary antibody (Beyotime) for 1 h at room temperature. Protein expression was assessed using an enhanced chemiluminescent substrate (Affinity) and exposed to a chemiluminescent film.

Colony formation assay

Colony formation was performed in 6cm culture dishes (Corning). Totally 400 cells were seeded per dish and cultured for 14 days. Colonies were fixed in methanol for 10min and stained with 0.1% crystal violet for 30min at room temperature. The number of colonies was counted by ImageJ.

Spheroid formation assay

100 cells were seeded into Costar Ultra Low Cluster 96-well plates (Corning) and cultured in PromoCell 3D Tumorsphere Medium XF according to the manufacturer's instructions.

Flow cytometry and cell sorting

After specific treatment, the cells were fixed with 4% paraformaldehyde for 20 min at room temperature. Then, they were incubated with 0.1% Triton X-100 in 1× PBS buffer for 15 min at room temperature and blocked with 3% BSA for 1 h at room temperature. Cells were stained with monoclonal antibodies for CD133, Kac, Kbhb or an isotype control antibody.

We used indirect staining for Kac and Kbhb detection. The cells were then incubated with pan-acylation antibodies (mouse Kac and rabbit Kbhb) provided by PTM for 1 h, washed three times with 1× PBS buffer, and centrifuged at 1000 rpm for 5 min each time. The diluted secondary antibodies were then added to the cells and incubated at room temperature for 1 h. After washing three times with 1× PBS buffer and centrifugation at 1000 rpm for 5 min each time, the cells were resuspended in 0.5 ml of PBS buffer and passed through a 400-mesh screen. Flow cytometric analysis was performed on Guava EasyCyte HT.

Flow cytometry cell sorting was conducted using PE-conjugated mouse anti-human CD133 (Miltenyi Biotec) and its respective isotype control, and then sorted on BD Aria III.

Fast protein liquid chromatography chromatography (FPLC)

PLC-PRF-5 cell extracts were applied to a Superdex 200 10/300 GL (GE Healthcare) equilibrated with PBS. The column was eluted at a flow rate of 0.5 mL/minute, and fractions were collected.

Co-IP assay

The cells were rinsed with cold PBS and lysed in IP lysis buffer (50 mM Tris-HCl, pH 8.0, 150 mM NaCl, 0.5% NP40), and the total protein in the lysate served as the "Input" sample. Then, protein A/G beads were incubated with specific antibodies (2–3 μg) or IgG separately for 6–8 h to form the immune complex. The beads were incubated with cell lysate overnight. The precipitated proteins were eluted from the beads by resuspending the beads in 2× SDS-PAGE loading buffer and boiling for 10 min. The resultant materials from immunoprecipitation or cell lysates were resolved using 10% SDS-PAGE gels and transferred onto PVDF membranes for Western blot analysis.

Duolink in situ proximity ligation assay (PLA)

The Duolink in situ PLA was performed using Duolink In Situ Red Starter Kit Mouse/Rabbit (Sigma-Aldrich) according to the manufacturer's protocol. In brief, PLC-PRF-5 cells and SUN-387 cells were plated on glass coverslips, rinsed three times with PBS and fixed in 4% formaldehyde in PBS for 10 min. The cells were permeabilized in 0.5% Triton X-100 for 5 min and blocked with 3% BSA in PBS for 60 min at 37 °C. After blocking, cells were then incubated with primary antibodies in PBS containing 1% BSA overnight at 4 °C, followed by incubation with corresponding secondary antibodies conjugated with PLA probes for 60 min at 37 °C in the dark. Cells were washed three times in wash buffer. Finally, the cells were stained with DAPI, and Duolink signals were detected using Nikon laser scanning confocal microscope A1 (Nikon, Japan).

ChIP

ChIP was performed with the Magna ChIP G-Chromatin Immunoprecipitation Kit (Cell Signaling Technology) according to manufacturer's instructions. Briefly, cells were crosslinked in the presence of 1% formaldehyde at room temperature for 10 min and harvested after washing with cold PBS. Immunoprecipitation of crosslinked protein/DNA was carried out with 4 mg of MTA2 antibody or rabbit IgG control. Immunoprecipitated and eluted DNA was purified with columns and amplified by qPCR.

DRIP

DRIP was performed as described in Stephan Hamperl et al., 2017.[2] The total nucleic acids of the cells were prepared using DNeasy Blood & Tissue Kits (QIAGEN) according to the manufacturer's instructions. DNA-RNA hybrids were immunoprecipitated from 2 μg of the total nucleic acids using S9.6 antibody (1 μg) overnight. The immunoprecipitate was purified and analyzed as ChIP samples.

Immunofluorescence staining

The cells were fixed with 4% formaldehyde in phosphate-buffered saline (PBS) for 5 min, permeabilized with 0.2% Triton X-100, blocked with 3% bovine serum albumin, and incubated overnight with primary antibodies at 4 °C. Alexa Fluor 488-labeled Goat Anti-Mouse IgG antibodies or Cy3-labeled Goat Anti-Rabbit IgG secondary antibodies (Beyotime) were incubated for 1 h at room temperature. Each step was followed by two washing procedures with for 5 min. Finally, the

cells stained with 4′,6-diamidino-2-phenylindole (DAPI; Beyotime) were mounted and viewed using a laser scanning confocal microscope A1 (Nikon, Japan). S9.6 intensity per nucleus was calculated using ImageJ, where DAPI is used as a mask for the nucleus, and the nuclear S9.6 signal intensity was then determined by subtracting the nucleolin signal and analyzing the intensity of the remaining S9.6 signal.[2, 3]

Luciferase Reporter Assay

Luciferase activity was measured using a dual luciferase reporter gene assay kit (Beyotime) according to the manufacturer's protocol. Each experiment was performed in triplicate and repeated at least three times.

Immunohistochemical (IHC) staining

IHC staining was performed using a two-step protocol as previously described. Briefly, after deparaffinized with xylene, rehydrated in graded ethanol, immersed in 0.3% hydrogen peroxide, and heat-mediated antigen retrieval in citric acid at pH 6.0, tissue section was incubated with the antibody for MTA2 (Bioss, bs-9440R) at 4 °C overnight, labeled by HRP (rabbit) second antibody (Thermo Scientific, USA) at room temperature for 60 min. Finally, sections were developed in DAB solution (Gene Tech, Shanghai, China) under microscopic observation and counterstained with hematoxylin. The scoring of expression was performed according to both of the ratio and intensity of positive-stained cells: 0–5% scored 0; 6–35% scored 1; 36–70% scored 2; and more than 70% scored 3. The final scores were designated as low or high expression as follows: low expression (score 0–1), high expression (score 2–3). These scores were determined independently by two experienced pathologists in a blinded manner, and mean percentage values were taken.

Animal studies

Animal experiments were conducted in accordance with the National Institutes of Health Animal Use Guidelines. All of the experimental protocols were approved by the Institutional Animal Care and Use Committee at Tianjin International Joint Academy of Biomedicine. Tumor-initiating and self-renewal abilities were investigated by limiting dilution and serial transplantation assays. 4 to 6-week-old male NOD/SCID mice were injected subcutaneously with 100, 1,000 or 10,000 cells. Tumor incidence and tumor latency were recorded. Tumor-initiating frequency was calculated using the Extreme Limiting Dilution Analysis (ELDA) software.[4]

For primary tumor growth and Kaplan–Meier survival analysis, a total of 1×10^7 cells were injected subcutaneously to BALB/C nude mice. When tumor volume reached approximately 50 mm^3, the diameters of the tumors and survival status were serially measured with a digital caliper every 3 days, and their volumes were calculated using the following formula: volume = (length × $width^2$)/2.

DEN-induced hepatocarcinogenesis: 3-week-old C57BL/6 mice were given a single intraperitoneal injection of DEN (50 μg/g body weight). One week later, the DEN-challenged mice were given intraperitoneal injections of adenovirus (1×10^8 plaque-forming units (pfu)) once every week until being killed after 10 months. The livers were then analysed for number and size of hepatic tumours.

Bioinformatics analysis

The clinical information and gene expression data of hepatocellular carcinoma in The Cancer Genome Atlas (TCGA) were downloaded using the R package “TCGAbiolinks”[5] and analyzed by the R package “ggplot2”, “survival” and “survminer”. Gene set enrichment analysis (GSEA) was carried out using R package “clusterProfiler”[6]. Gene co-expression analysis and correlation analysis were performed using the R package “ggstatsplot” and “corrplot”.

Patient samples and ethics

We complied with all relevant ethical regulations. We collected 340 paraffin-embedded specimens of patients with HCC and analyzed the clinical data integrity. These cases were from Tianjin Medical University General Hospital, Tianjin Cancer Hospital, Shandong Shouguang Hospital, and Affiliated Hospital of Jining Medical University.

Statistical analysis

Statistical analyses were performed using GraphPad Prism version 8 for Windows or R 3.6.0. Statistically significant differences were calculated using Student’s *t*-test, one-way ANOVA, Pearson’s correlation, and Kaplan–Meier as needed. $P < 0.05$ was considered significant.

DISCUSSION

The roles of MTA2 in the progression of malignant tumors have been reported extensively [7]. However, previous studies prioritized phenomics. The MTA2 expression levels in cases with HCC are related to the malignant progression of HCC [8]. MTA2 can inhibit the Hippo pathways, thereby promoting the development of HCC.[9] In general, after being recruited by the transcription factor, MTA2, as a component of the NURD complex, can induce transcriptional inhibition by combining the promoter of the downstream target gene. For instance, the key transcription factor TWIST that drives the epithelial–mesenchymal transition (EMT) can recruit the NURD complex, transcriptionally inhibit CDH1, promote EMT in cancers, and stimulate the progression of malignant tumors [10]. The oncogenic transcription factor PML-RARα is detected in human acute promyelocytic leukemia, and it can recruit the NuRD to bind, transcribe, and inhibit the target gene RARβ2, which is a tumor suppressor gene [11]. In pancreatic carcinoma, HIF-1α can also recruit the NURD complex and transcriptionally inhibit CDH1 [12]. All these mechanisms depend on the motifs of the transcription factors, and the target gene is also specific. A new molecular mechanism was established in the present study., With the incomplete NURD complex, MTA2 was independent of the transcription factor and could be directly induced to form the R-loop to exert transcriptional inhibition, and the target genes were completely different from the transcriptional regulation that depended on the transcription factor. Thus, the phenotypes differed. MTA2 drives the stemness enhancement of tumor cells through the R-loop pathway, which is a new feature of MTA2 that has never been reported.

The DNA-RNA hybrid chain (R loop) exists, and the functions of R loop vary with conditions. The R-loop that is formed in a physiological manner on the CpG island where the gene promoters are enriched can protect the genes from preferential methylation and silencing [13]. The R-loop is also enriched at a G-rich terminator element, which helps suspend RNA polymerase II before it

effectively terminates [14]. Thus, the R-loop can regulate gene expression, but research on this field is extremely limited. The R-loop that is involved in many physiological processes can cause DNA damage. The mechanism underlying the regulation of DNA damage caused by the R-loop is a topic that has received the most in-depth research in the field of R-loop [15]. Given that the R-loop leads to genomic instability and replication stress, which are also features of tumor cells, the R-loop is associated with tumors. However, studies that directly explain the relationship between R-loop and tumors are limited [16]. The relationship between the R-loop and HCC has not been reported yet. The R-loop may be one of the mechanisms of cellular self-regulation to avoid overactive transcription and replication. Many RNA-binding proteins can induce the formation of the R-loop and include RNA processing proteins, helicase, histone-modifying enzymes, and DNA repair proteins [17]. A total of 252 (47%) of the 536 proteins that were identified in the present study had poor prognosis in HCC, which may indicate an association between the R-loop and cancers. MTA2 directly promoted the formation of R-loop, and the MTA2-induced R-loop could specifically regulate the expression levels of several genes (e.g., BDH1), thereby inhibiting the transcription of the genes and downregulating the levels of mRNA and protein. Sites that can form r-loops are widespread [2]. Although many sites can form the R-loop, proteins that induce the formation of the R-loop are likely to be the key to provide the specificity of the R-loop. The MTA2-induced R-loop proves this point. Only when an incomplete NURD complex is recruited will MTA2 induce the R-loop. This result may be due to the weak ability of MTA2 itself to recruit transcription factors and to the lack of the transcription factors required for the microenvironment for the nuclei of the HCC cells.

A total of 96 genes were obtained by screening the signals inside the open reading frames of S9.6 RDIP-seq and MTA2 ChIP-seq. Among the genes, the only one with a significant co-expression relationship with MTA2 and CD133 and a significant impact on the overall survival of HCC was BDH1. BDH1 encodes 3-hydroxybutyrate dehydrogenase 1, which is a key enzyme in the metabolic process of ketone bodies, and it controls the transformation between the ketone bodies AcAc and βHB. The relationship between BDH1 and cancers is rarely studied. However, the relevant TCGA data show that BDH1 is a good prognostic marker for various types of tumors (including HCC) and a tumor suppressor gene. The balance of the two ketone bodies controlled by BDH1 plays an important role in the formation and progression of HCC. The reprogramming of energy metabolism is also a common feature of cancer [18]. The formation and progression of HCC are often accompanied with the reprogramming of metabolic pathways, resulting in a metabolic disorder [19]. The liver is the most important metabolic organ. Fatty liver is a high-risk factor and a necessary condition for HCC. Changes in lipid metabolism may also lead to the accumulation of fatty acid and lipid, which do not only promote the development of fatty liver but also provide a microenvironment for the occurrence and progression of HCC [20]. In particular, ketone bodies, as the body's energy source when hungry, are signaling molecules themselves [21]. The main substrate of ketogenesis is fatty acid. Any abnormal lipid metabolism of HCC may inevitably result in abnormal ketogenesis. Ketogenic dysfunction can cause adverse effects on the liver [22]. Ketone bodies promote the formation [23] and metastasis [24] of tumor stem cells, but the mechanism is unclear. Ketone bodies are mainly produced in the liver. Hepatocytes do not have key enzymes required for the use of ketone bodies [21]. However, researchers have found that HCCs produce key enzymes required for use of ketone bodies, which promote the development of HCC [25].

The mechanism of transcriptional regulation of BDH1 has not been identified yet. The transcription factor BMAL1 in the heart can directly inhibit the transcription of BDH1 [26]. The

transcription factor E2F6 in the heart can also activate BDH1 expression [27]. These studies are all regulation modes of BDH1, which is related to the transcription factor. In the present study, a trans-regulatory mechanism was suggested, which was independent of the transcription factor and brought by the R-loop induced by an incomplete NURD complex. In the microenvironment for HCC cells, many normal functions of cells are disordered at hunger, requiring many NURD complexes for histone deacetylation. When a complete complex is difficult to form due to the lack of components, an incomplete NURD complex may be produced. The "emergency" mechanism of the R-loop that is independent the transcription factor performs transcriptional inhibition, and the target gene is BDH1. This mechanism can make the ketone body βHB be produced in a large quantity as a hunger signal, thereby promoting stemness.

Yingming Zhao's group employed mass spectrometry to discover many types of acetylation on lysine, which mostly came from human metabolites, including βHB. [28] They showed that the βHB level increases during starvation, which can induce lysine Kbhb. [29] They also revealed that the enrichment of the histone H3K9bhb at the promoter is close to and positively correlated with the chromatin accessibility markers H3K9ac and H3K4me3, thereby proving that H3K9bhb is a transcriptional activation marker. For a hunger-induced highly-expressed gene, H3K9bhb and H3K4me3 are highly expressed, and H3K9ac is poorly expressed at TSS. Therefore, the genes regulated by H3K9bhb are microenvironmentally specific, and their effects of opening chromatin can differ from that of H3K4me3 and H3K9ac. They also pointed out that hunger induction can significantly upregulate cancer-related pathways, which are also closely related to H3K9bhb [29]. Given that the enhancement of stemness requires DNA demethylation and heterochromatin opening [30], H3K9bhb is a "super switch" in a heterochromatin opening-specific region. In the present study, any downregulation of BDH1 could increase the Kbhb and H3K9bhb levels, causing changes in the chromatin state and transcriptome. After contact with Zhao's group, we got the list of H3K9bhb-regulated genes identified in mice. Most of the genes are homologous to humans. The roles of MTA2-induced R-loop, BDH1, and βHB on the genes in HCC cells were identified. The obtained results were consistent with Zhao's study and proved that the metabolite βHB could enhance the stemness of HCC via H3K9bhb.

Our work has certain limitations. This study was limited to HCC, but the investigated molecular mechanism may be prevalent, which needs to be further verified in additional tests. While investigating Kbhb, H3K9bhb that has chromatin remodeling functions was chosen. Kbhb also has unique features in other proteins or at other sites, which should be further studied in future work.

As revealed in the present study, MTA2 could transcriptionally inhibit BDH1 by recruiting the incomplete NURD complex-induced R-loops, thereby leading to the accumulation of βHB, an increase in H3K9bhb, and a waterfall effect on HCC formation and progression. The abnormal metabolism and microenvironment of the organism are also important conditions for tumor formation and progression.

Supplemental Figures

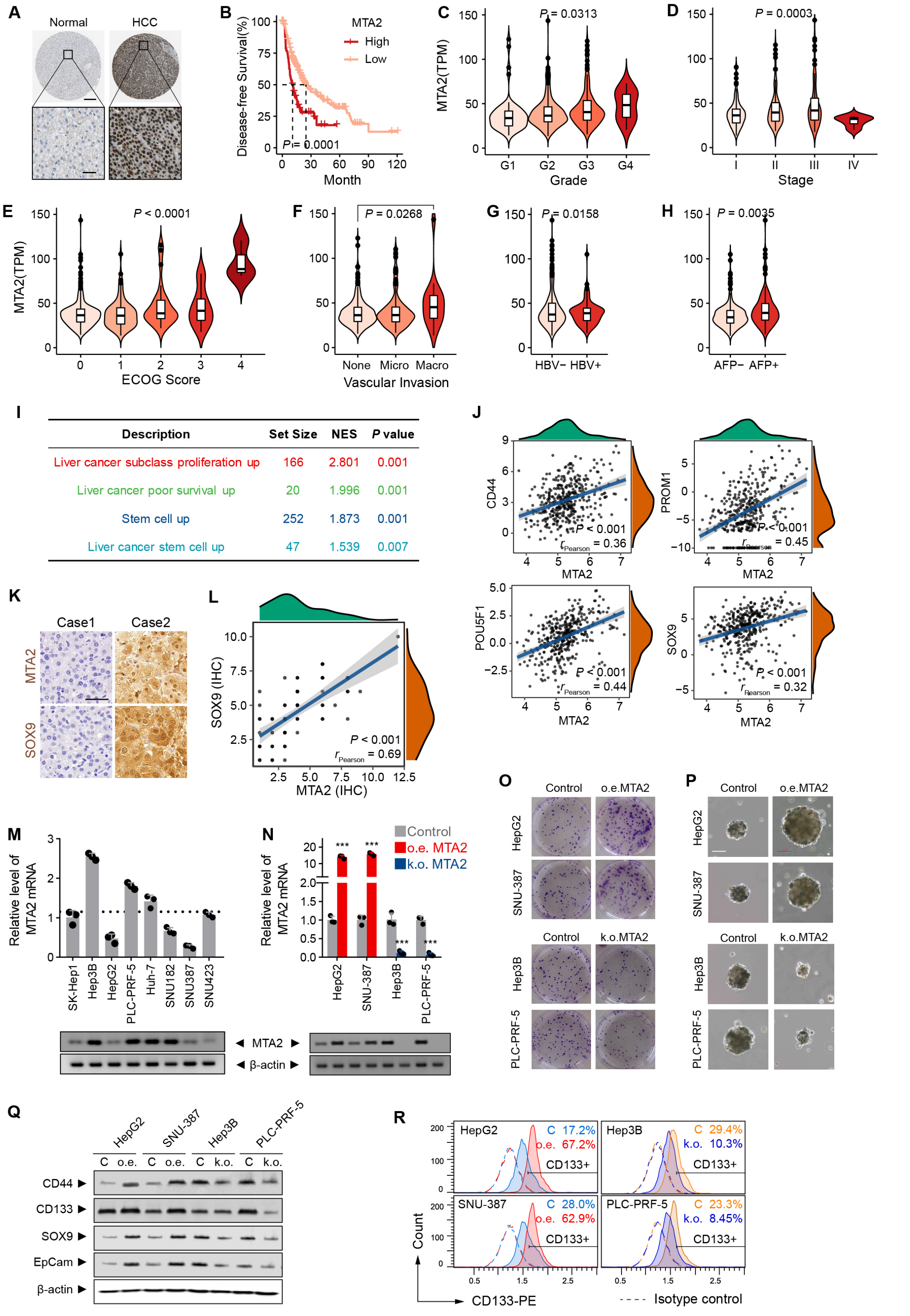

Fig. S1 MTA2 promotes stemness properties in HCC

(A) Immunohistochemical staining of MTA2 in adjacent tissues and HCC tissues in Human Protein Atlas. Upper scale bar: 200 μm, lower scale bar: 40 μm.

(B) Kaplan–Meier curves showing percentage of the disease-free survival of the higher and lower expression of MTA2.

(C-F) MTA2 mRNA expression level (TPM) in different Grade(C), Stage(D), ECOG score(E) and Vascular patients in the TCGA dataset. One-way ANOVA and Tukey's multiple comparisons test was used.

(G-H) MTA2 mRNA expression level (TPM) in different HBV status(G) and APF status(H) patients in the TCGA dataset. Student's *t*-test was used.

(I) GSEA analysis details of patients with high or low MTA2 expression.

(J) Co-expression analysis of MTA2 expression and stem cell markers CD44 (G), PROM1 (CD133, H), POU5F1 (Oct4, I), and SOX9(J) in the TCGA dataset.

(K-L) Representative image of MTA2 and SOX9 immunohistochemistry(K), and statistical analysis of the immunoreactive score confirmed that MTA2 was positively correlated with the expression of SOX9(L). Scale bar: 40μm.

(M) Quantification of MTA2 mRNA (upper) and protein (lower) in 8 HCC cell lines. n=3, biological replicates.

(N) MTA2 overexpression and knockout were performed in 4 HCC cell lines, and the levels of MTA2 mRNA (upper) and protein (lower) were detected. ***p<0.001, Student's *t*-test. n=3, biological replicates.

(O-P) Representative image of plate colony formation (O) and oncosphere formation (P) obtained after overexpressing or knocking out MTA2 in four HCC cell lines. Scale bar in (P): 100 μm. n=3, biological replicates.

(Q) Western blot of stem cell markers CD44, CD133, SOX9, and EpCam after overexpression or knockout of MTA2 in four HCC cell lines.

(R) Flow cytometry for CD133 after overexpression or knockout of MTA2 in four HCC cell lines.

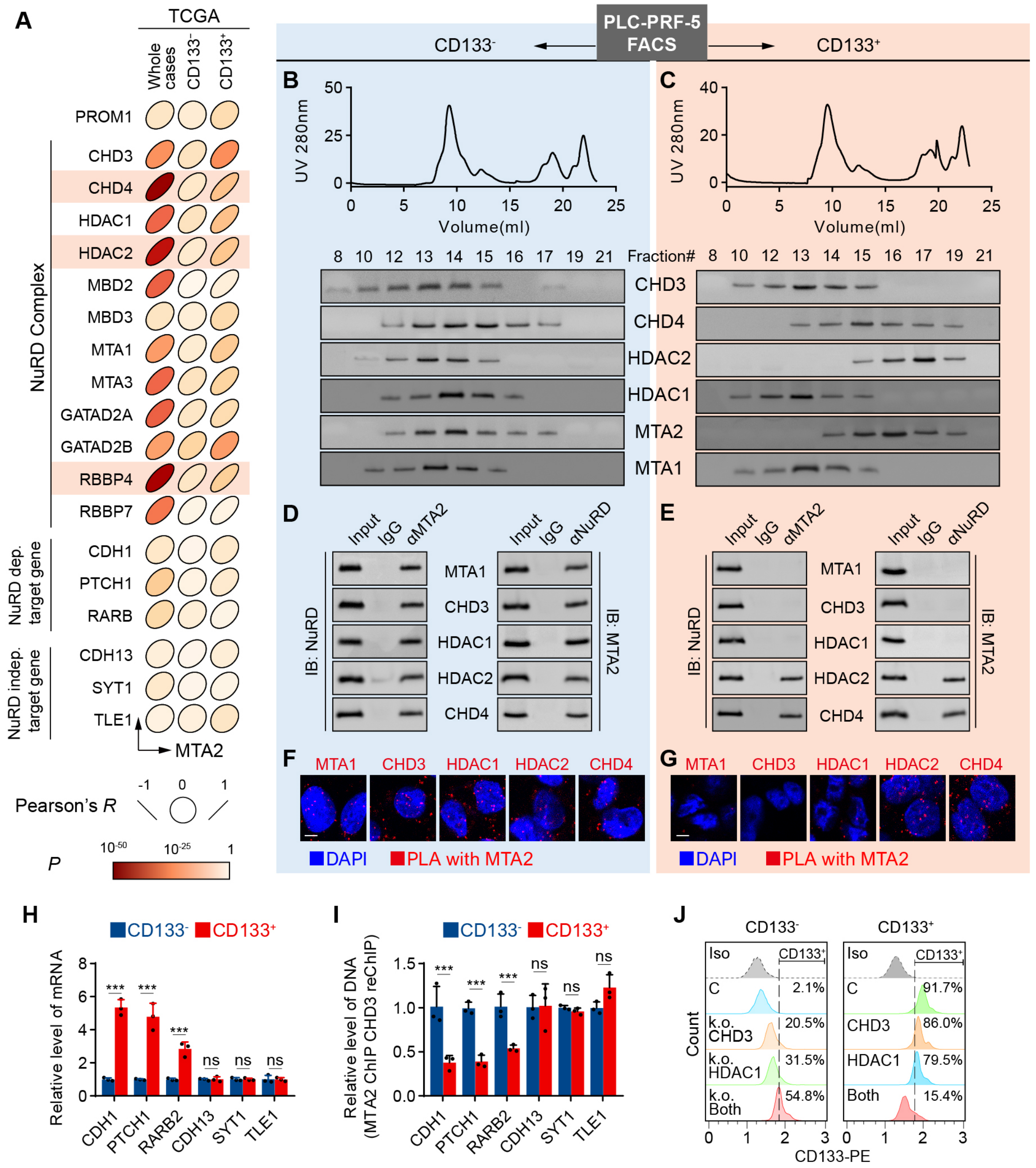

Fig. S2 MTA2 recruits HDAC2/CHD4 in CD133$^+$ HCC cells

(A) Correlation analysis between the expression of MTA2 and the components of the NuRD complex, the NuRD complex-dependent target genes, and the NuRD complex-independent MTA2 target genes in the TCGA LIHC whole cases, CD133$^-$ and CD133$^+$ cases. The closer the ellipse is to a straight line, the closer the Pearson correlation coefficient R value is to ± 1, and the color depth represents the P value. See also Table S2.

(B-C) Fast protein liquid chromatography experiments in CD133$^-$ (C) and CD133$^+$ (D) cells. Chromatographic elution profiles and IB of the chromatographic fractions are shown. Equal volume from each fraction was analyzed.

(D-E) Co-immunoprecipitation assays in CD133$^-$ (D) and CD133$^+$ (E) cells with anti-MTA2 followed by immunoblotting with antibodies against the indicated proteins or with antibodies against the indicated proteins followed by IB with anti-MTA2.

(F-G) PLA assays in CD133$^-$ (F) and CD133$^+$ (G) cells with MTA2 and indicated proteins. Scale bar: 5 μm.

(H) qPCR results of the mRNAs of NuRD complex-dependent target genes and NuRD complex-independent MTA2 target genes in CD133$^-$ and CD133$^+$ PLC-PRF-5 cells. ***p<0.001, Student's *t*-test. n=3, biological replicates.

(I) Results of qPCR after MTA2 ChIP and CHD3 reChIP of NuRD complex-dependent target genes and NuRD complex-independent MTA2 target genes in $CD133^-$ and $CD133^+$ PLC-PRF-5 cells. ***p<0.001, Student's *t*-test. n=3, biological replicates.

(J) Flow cytometry for CD133 after overexpression or knockout of indicated genes in $CD133^-$ and $CD133^+$ PLC-PRF-5 cells.

The experiments in (I) and (J) were conducted to investigate whether there is a balance shift between the two complexes and whether the corresponding function changes if other components of NuRD are supplemented. The results showed that after supplementing HDAC1 and CHD3, MTA2 functions mainly in the form of NuRD complex binding to DNA, and not in the R-loop pathway.

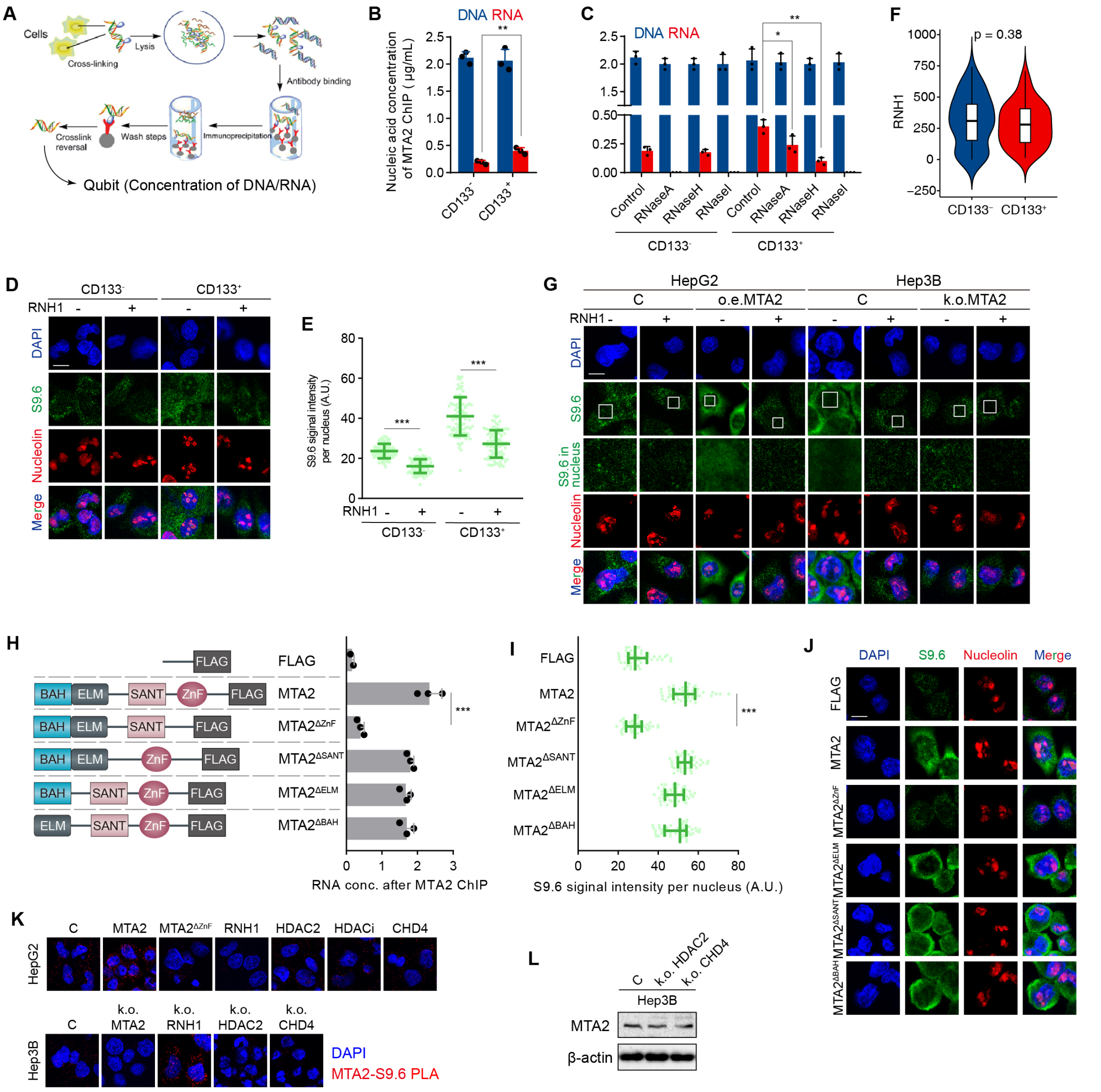

Fig. S3 MTA2-triggered R-loop in CD133^{+} HCC cells

(A) Illustration of ChIP experiment.

(B) Concentrations of DNA and RNA after MTA2 ChIP in CD133^{-} and CD133^{+} PLC-PRF-5 cells. n=3, biological replicates.

(C) Concentrations of DNA and RNA after MTA2 ChIP and different ribonuclease treatments in CD133^{-} and CD133^{+} PLC-PRF-5 cells. 0.3 M NaCl concentration was used for RNase A treatment to ensure that it can specifically cleave single-stranded RNA. n=3, biological replicates.

(D-E) Immunostaining(D) and quantification(E) of S9.6 nuclear signal in CD133^{-} and CD133^{+} PLC-PRF-5 cells. The nucleus was co-stained with nucleolin antibody and treated with RNaseH as indicated. A.U., arbitrary units. The median of the S9.6 signal intensity per nucleus after nucleolar signal removal is shown.

(F) The expression of RNaseH in the two populations of stem cells with different stemness properties marked by CD133. (from single-cell sequencing data GSE103866)

(G) Immunostaining of S9.6 nuclear signal after overexpression or knockout of MTA2 in four HCC cell lines. The nucleus was co-stained with nucleolin antibody and treated with RNase H1 as indicated.

(H) RNA concentration after FLAG ChIP in indicated PLC-PRF-5 cells. n=3, biological replicates.

(I-J) Immunostaining (J) and quantification(I) of S9.6 nuclear signal in indicated PLC-PRF-5 cells. A.U., arbitrary units. The median of the S9.6 signal intensity per nucleus after nucleolar signal removal is shown.

(K) MTA2 and S9.6 antibodies were used for PLA to perform confocal imaging of MTA2-induced R-loop in two HCC cell lines. Scale bar: 10 μm. HDACi (HDAC inhibitor): SAHA.

(L) Western blot analysis showed that HDAC2 or CHD4 knockout in Hep3B cells did not affect the expression of MTA2.

*p<0.05, **p<0.01, ***p<0.001, Student's *t*-test.

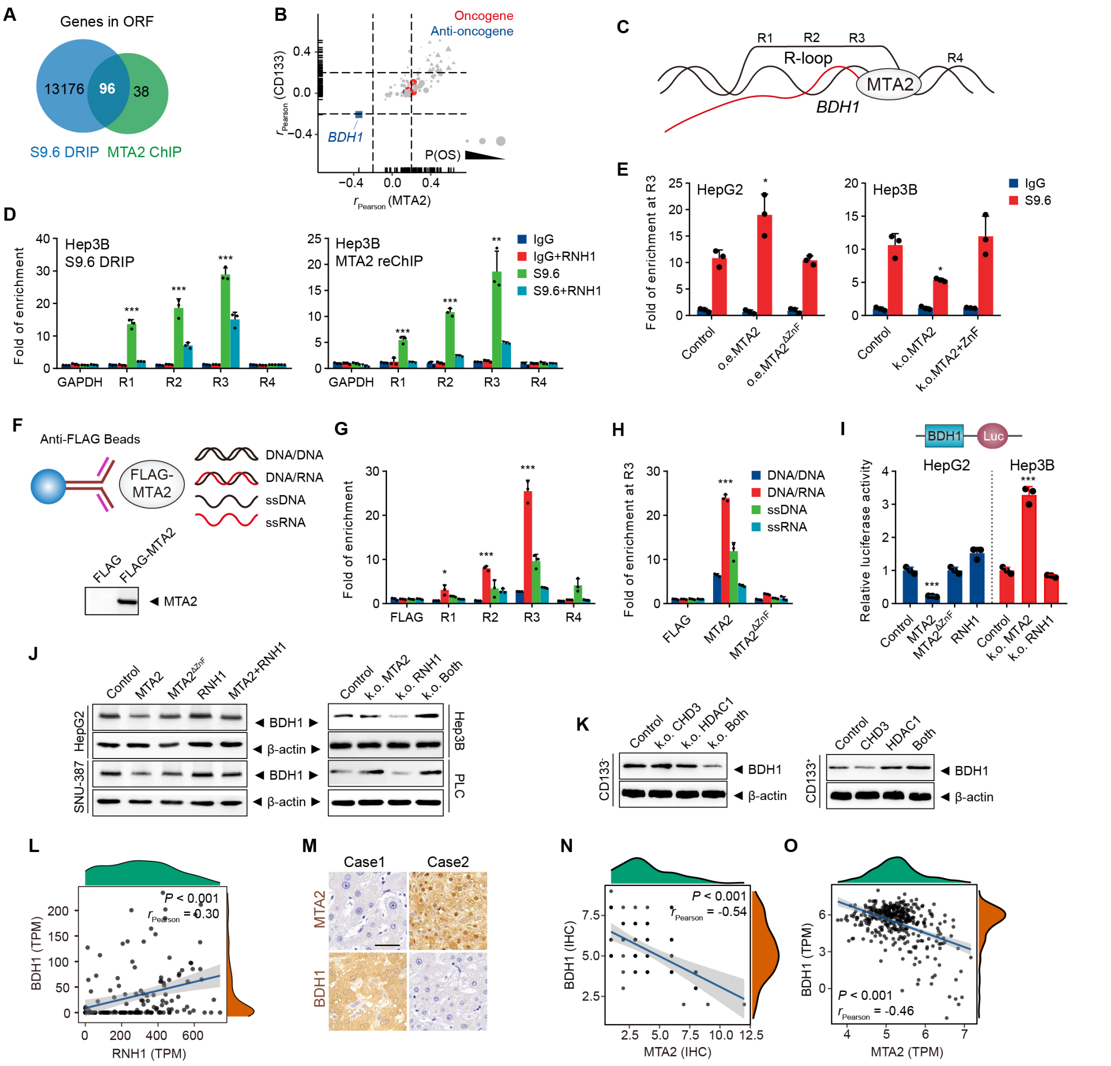

Fig. S4 BDH1 is transrepressed by MTA2-triggered R-loop

(A) Venn diagram of genes in open reading frames from S9.6 RDIP and MTA2 ChIP.

(B) Pearson correlation coefficients of the 96 intersecting gene expressions in Figure A, MTA2 and CD133, and the P value of the overall survival KM analysis.

(C) Schematic diagram of 4 regions of *BDH1* gene.

(D) Left: DRIP-qPCR using the anti-RNA–DNA hybrid S9.6 monoclonal antibody in Hep3B cells at four regions of *BDH1* gene and GAPDH. Right: S9.6 DRIP and MTA2 reChIP-qPCR in Hep3B cells at four regions of *BDH1* gene and GAPDH. n=3, biological replicates.

(E) DRIP-qPCR using the anti-RNA–DNA hybrid S9.6 monoclonal antibody in the overexpression (left) or knockout (right) of MTA2 in cells at R3. n=3, biological replicates.

(F) Schematic diagram and system verification of the in vitro IP experiment.

(G) Binding properties of MTA2 at four regions of *BDH1* gene obtained by in vitro IP-qPCR with different forms of nucleic acids. n=3, biological replicates.

(H) Binding properties of FLAG, FLAG-MTA2, or FLAG-MTA2$^{\Delta ZnF}$ at R3 of the *BDH1* gene obtained by in vitro IP-qPCR with different forms of nucleic acids. n=3, biological replicates.

(I) Up: the schematic illustration of luciferase reporter gene plasmid. Down: Luciferase reporter assays were performed to compare the luciferase activity of BDH1 in two HCC cell lines. n=3, biological replicates.

(J) Western blot of BDH1 protein after indicated treatments in two HCC cell lines.

(K) Western blot of BDH1 protein after indicated treatments in $CD133^-$ and $CD133^+$ PLC-PRF-5 cells.

(L) Co-expression analysis of BDH1 expression and RNH1 from single-cell sequencing data GSE103866.

(M-N) Representative image of MTA2 and BDH1 immunohistochemistry(H), and statistical analysis of the immunoreactive score confirmed that BDH1 was negatively correlated with the expression of MTA2(I). Scale bar: 40μm.

(O) Co-expression analysis of MTA2 expression and BDH1 in TCGA dataset.

*p<0.05, **p<0.01, ***p<0.001, Student's *t*-test.

On the basis of the regulatory region in Fig. 1h, four regions are selected and shown in Fig. S4C. The strength of the R-loop in the four regions is verified by DRIP-qPCR in the Hep3B cell line. The result shows that R-loop binding signals are identified by S9.6 DRIP in R1, R2, and R3, with R3 having the strongest signal (Fig. S4D, left). Almost no R-loop binding signal can be observed in the R4 region. This result is in good agreement with the ChIP-seq peak of MTA2 in the BDH1 reading frame. We further captured the DNA/RNA heterozygous double strands by using the S9.6 antibody and then verified whether MTA2 binding R-loops exist in these regions by using the MTA2 antibody (Fig. S4D, right). MTA2-binding R-loops are identified in R1, R2, and R3, with R3 having the strongest signal. This finding indicates that R3 is the main region under the regulation of the R-loop induced by MTA2. We further studied whether the R-loop level in this region depends on the expression of MTA2. By conducting qPCR in the R3 region after S9.6 DRIP, we found that after the overexpression of MTA2, the level of R-loops in R3 increases, whereas the overexpression of mutant MTA2 lacking the RNA binding domain ZnF cannot increase the level of R-loops in R3. After the knockout of MTA2, the R-loops formed in R3 decrease correspondingly. A simple overexpression of the ZnF domain can also increase the R-loop level of R3 (Figs. S4E). An in vitro experiment was also designed (Fig. S4F) to verify whether MTA2 can bind DNA/RNA heterozygous double strands in the four regions and realize the sequence affinity of the MTA2-binding R-loop. DNA and RNA corresponding to the R1–R4 regions were synthesized. DNA double strands and DNA–RNA heterozygous double strands were obtained through annealing. At the same time, DNA sense strand and RNA antisense strand were used as control. As shown in Fig. S4G, the corresponding nucleic acid chain and FLAG-MTA2 protein expressed by 293 cells were added into the system, and then the FLAG antibody was used for immunoprecipitation. MTA2 can be detected from the product obtained through this method, indicating that this system is effective. After the nucleic acid captured by MTA2 was quantitatively detected by qPCR, the result obtained is similar to those of intracellular experiments; specifically, the binding signals were detected in groups where R1, R2, and R3 were added into the DNA–RNA heterozygous double strands, where the signal of R3 was the strongest. In addition, the single-stranded DNAs of R1, R2, and R3 also had weaker signals than those of the DNA–RNA heterozygous double strands, where the signal of R3 was relatively strong. A weak signal also appeared in the single-stranded RNA of R3, whereas no specific signal was found in other groups. This result shows that MTA2 has the ability to bind specific DNA–RNA heterozygous double strands strongly and single-stranded DNA and RNA weakly (Fig. S4G). Taking R3 with the strongest binding as an example, we further studied whether this binding ability is related to the ZnF domain. As shown in Fig. S4H, when MTA2 is replaced by the mutant FLAG-$MTA2^{\Delta ZnF}$ group, the binding ability to four nucleic acids is almost lost.

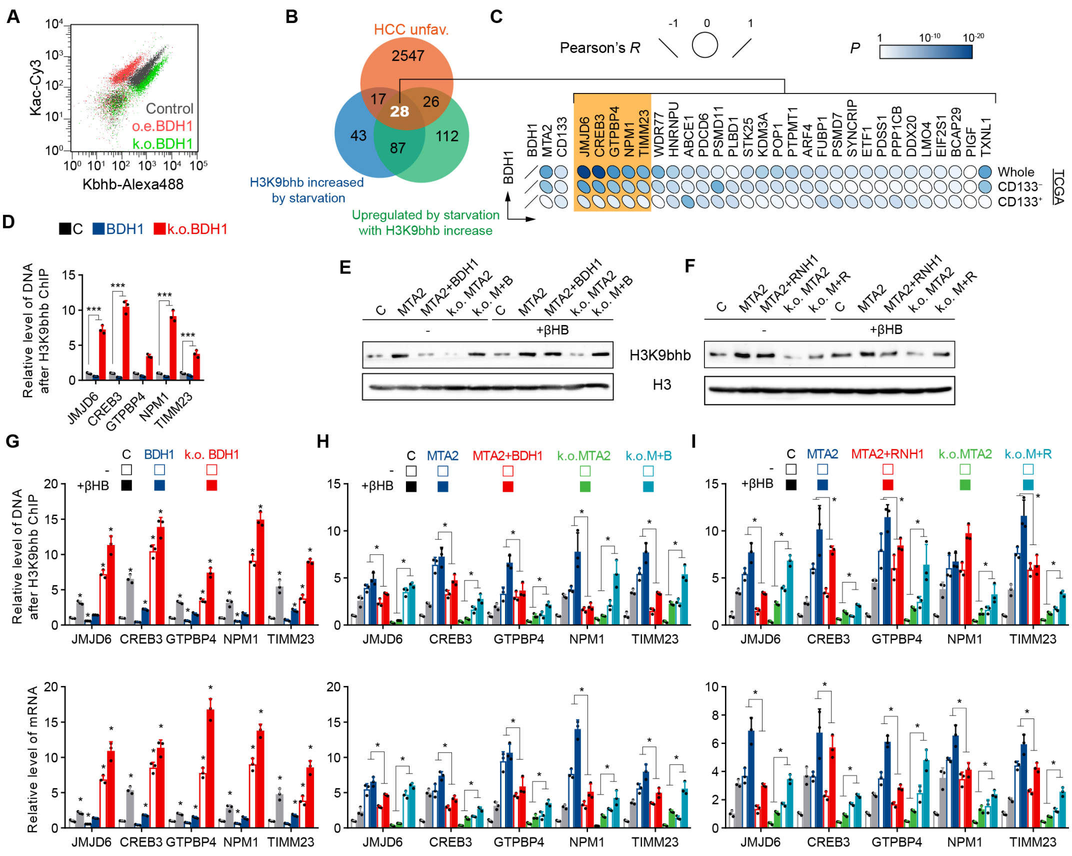

Fig. S5 βHB accumulation due to BDH1 deficiency increases Kbhb, especially H3K9bhb

(A) Flow cytometry for Kac and Kbhb after overexpression or knockout of BDH1 in PLC-PRF-5 cells.

(B) Venn diagram of HCC unfavorable genes, H3K9bhb increased by starvation genes, and upregulated genes with increasing H3K9bhb.

(C) Correlation analysis between the expression of BDH1 and the 28 intersecting genes in (D) in the TCGA LIHC whole cases, $CD133^-$ and $CD133^+$ cases. Orange marks five unfavorable genes with the most significant negative correlation with BDH1 in the $CD133^+$ cases. The closer the ellipse is to a straight line, the closer the Pearson correlation coefficient R value is to ± 1, and the color depth represents the P value. See also Table S4.

(D) H3K9bhb ChIP-qPCR at five genes filtered in (E) after overexpression or knockout of BDH1 in PLC-PRF-5 cells. ***$p<0.001$, one-way ANOVA and Tukey's multiple comparisons test. n=3, biological replicates.

(E-F) Western blot of H3K9bhb on the condition that MTA2, BDH1(E), RNH1(F) were overexpressed or knocked out in PLC-PRF-5 cells. ***$p<0.001$, one-way ANOVA and Tukey's multiple comparisons test.

(G-I) H3K9bhb ChIP-qPCR(up) and qRT-PCR(down) of five genes filtered in (E) on the condition that the precursor βHB were supplemented (G), and MTA2, BDH1(H), RNH1(I) were overexpressed or knocked out in PLC-PRF-5 cells. *$p<0.05$, one-way ANOVA and Tukey's multiple comparisons test. n=3, biological replicates.

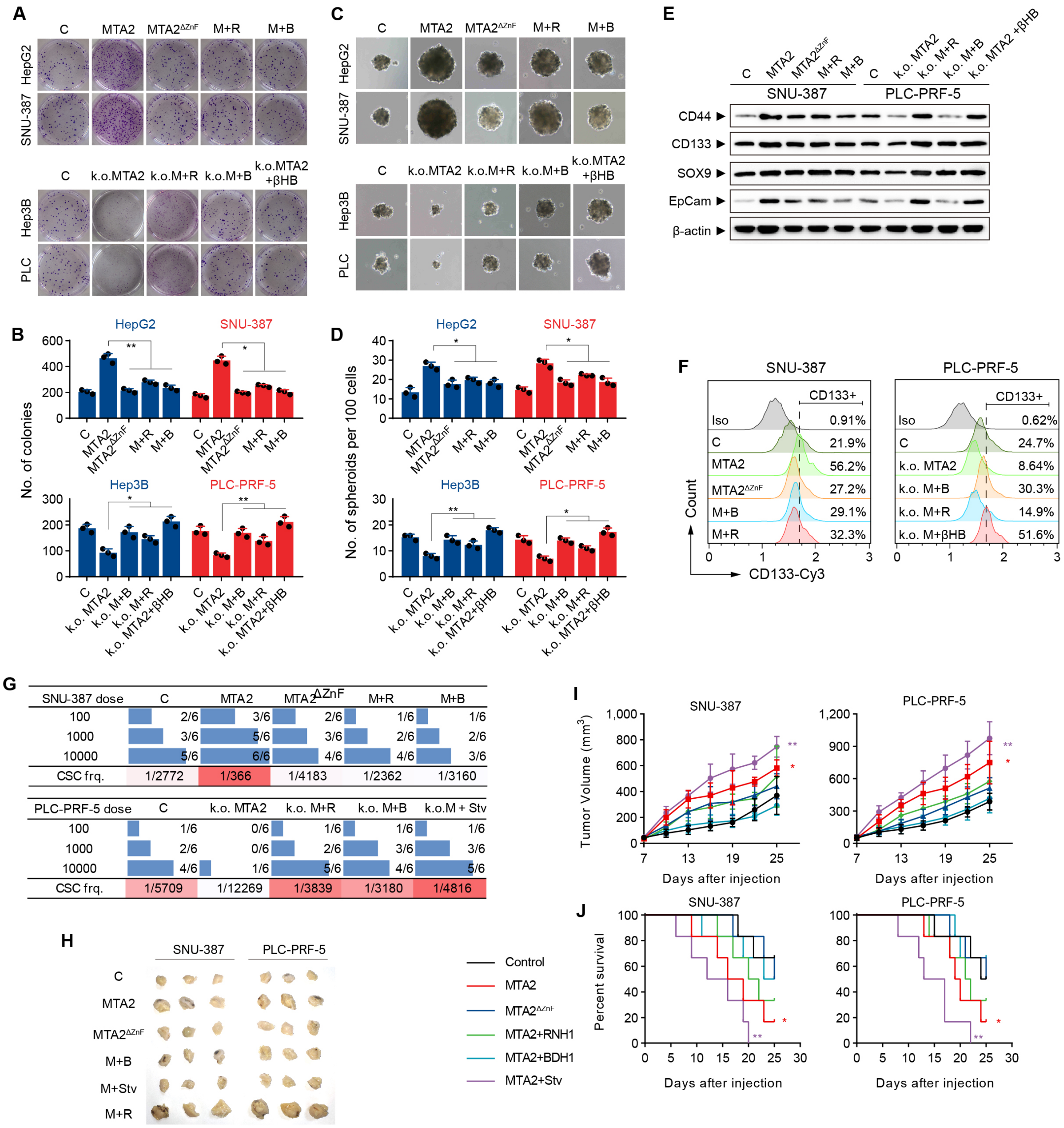

Fig. S6 MTA2-Rloop-BDH1-Kbhb axis plays an important role in HCC stemness in vitro and in vivo

(A-B) Representative image of plate colony formation (A) and quantification(B) after indicated treatments in four HCC cell lines. *p<0.05, **p<0.01, Student's *t*-test. n=3, biological replicates.

(C-D) Representative image of oncosphere formation (C) and quantification(D) after indicated treatments in four HCC cell lines. *p<0.05, **p<0.01, Student's *t*-test. n=3, biological replicates.

(E) Western blot of stem cell markers CD44, CD133, SOX9, and EpCam after indicated treatments in four HCC cell lines.

(F) Flow cytometry for CD133 after indicated treatments in four HCC cell lines.

(G) Limiting dilution assay was performed to compare the frequency of stem cells (SC) after indicated treatments in two HCC cell lines. n=6, biological replicates.

(H) Primary tumors of mice after indicated treatments.

(I) Primary tumor growth of mice was measured starting 7 days after implantation of SNU-387 and PLC-PRF-5 cells after indicated treatments. n=6, biological replicates.

(J) Kaplan–Meier curves showing percentage of survival of mice after implantation SNU-387 and PLC-PRF-5 cells after indicated treatments. *p<0.05, **p<0.01.

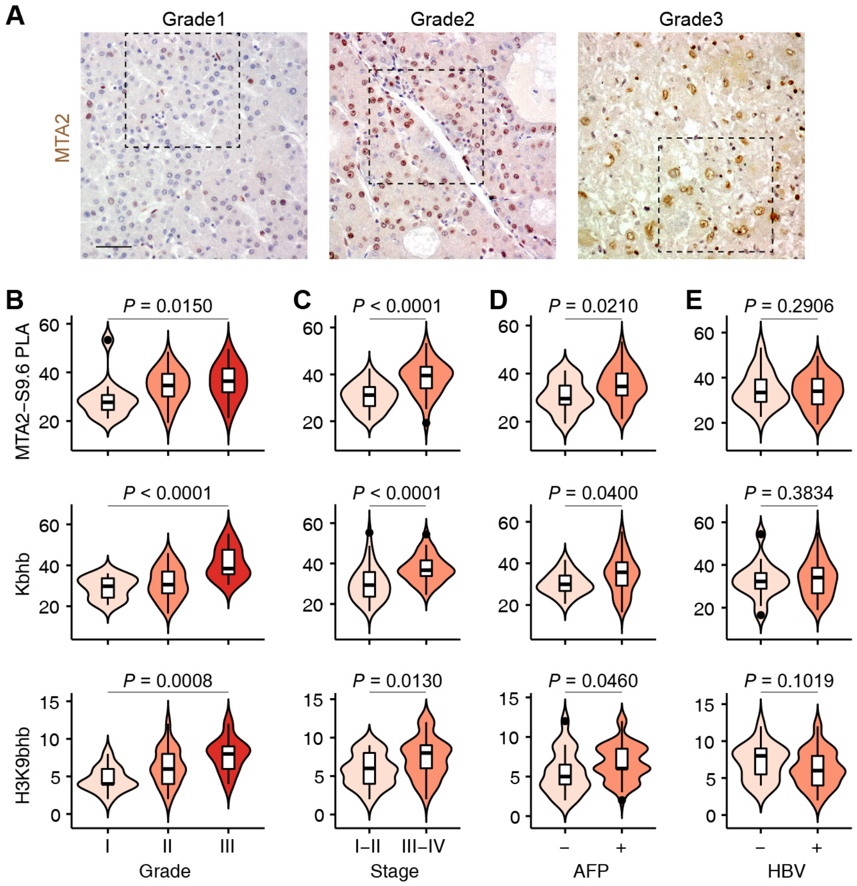

Fig. S7 MTA2-triggered R-loop, Kbhb and H3K9bhb can be used as prognostic markers for HCC

(A) Representative MTA2 IHC image of different grade HCC cases. Scale bar: 50 μm.

(B) Relationship between MTA2-S9.6 PLA, Kbhb, H3K9bhb signal and grade. One-way ANOVA and Tukey's multiple comparisons test was used.

(C-E) Relationship between MTA2-S9.6 PLA, Kbhb, H3K9bhb signal and clinical stage (C), AFP level (D) and HBV(E). Student's *t*-test was used.

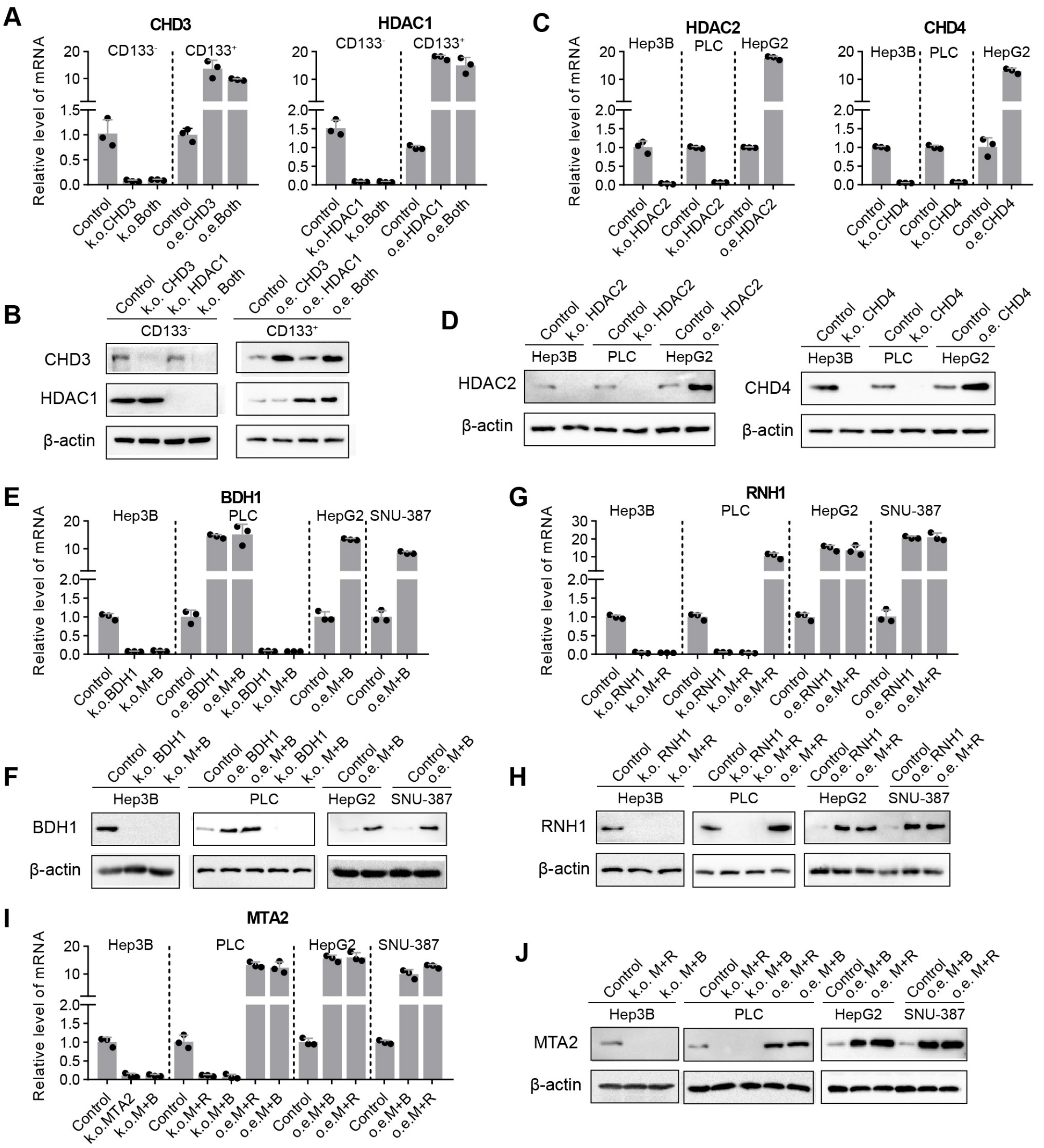

Fig. S8 Validation of overexpress and knockout cells

(A) The levels of CHD3 and HDAC1 mRNA were detected in indicated overexpression and knockout cells. n=3, biological replicates. k.o. Both: k.o.CHD3 and k.o. HDAC1. o.e. Both: o.e. CHD3 and o.e. HDAC1.

(B) The levels of CHD3 and HDAC1 protein were detected in indicated overexpression and knockout cells. k.o. Both: k.o.CHD3 and k.o. HDAC1. o.e. Both: o.e. CHD3 and o.e. HDAC1.

(C) The levels of CHD4 and HDAC2 mRNA were detected in indicated overexpression and knockout cells. n=3, biological replicates.

(D) The levels of CHD4 and HDAC2 protein were detected in indicated overexpression and knockout cells.

(E) The levels of BDH1 mRNA were detected in indicated overexpression and knockout cells. n=3, biological replicates.

(F) The levels of BDH1 protein were detected in indicated overexpression and knockout cells.

(G) The levels of RNH1 mRNA were detected in indicated overexpression and knockout cells. n=3, biological replicates.

(H) The levels of RNH1 protein were detected in indicated overexpression and knockout cells.

(I) The levels of MTA2 mRNA were detected in indicated overexpression and knockout cells. n=3, biological replicates.

(J) The levels of MTA2 protein were detected in indicated overexpression and knockout cells.

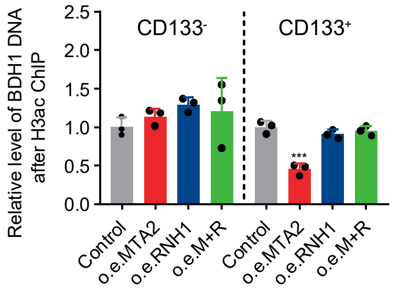

Fig. S9 MTA2 induced the R-loop and recruited HDAC2 to deacetylate histones at BDH1 in CD133⁺ HCC cells ***p<0.05, Student's *t*-test.

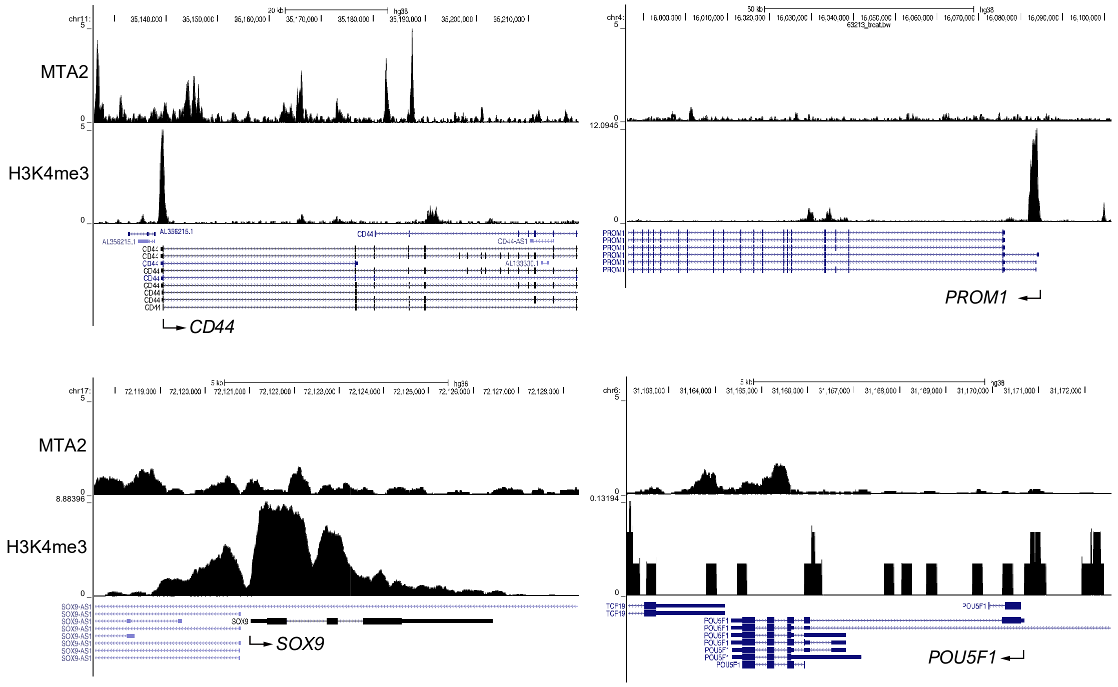

Fig. S10 MTA2 ChIP-seq data showed that MTA2 did not bind the promoter of CD44, CD133(PROM1), SOX9, and Oct4(POU5F1).

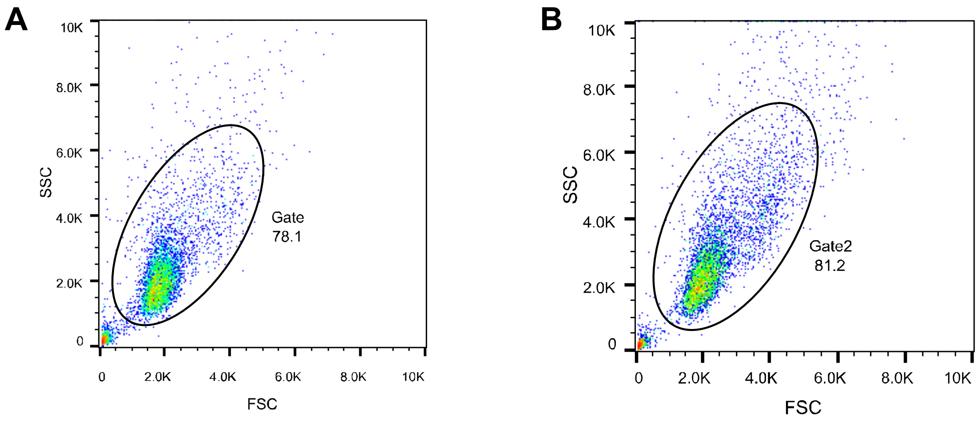

Fig. S11 Gating strategy for CD133(A) and Kac/Kbhb(B).

Main cell gate were determined by FSC and SSC, and the obtained signal higher than the isotype part is considered as positive.

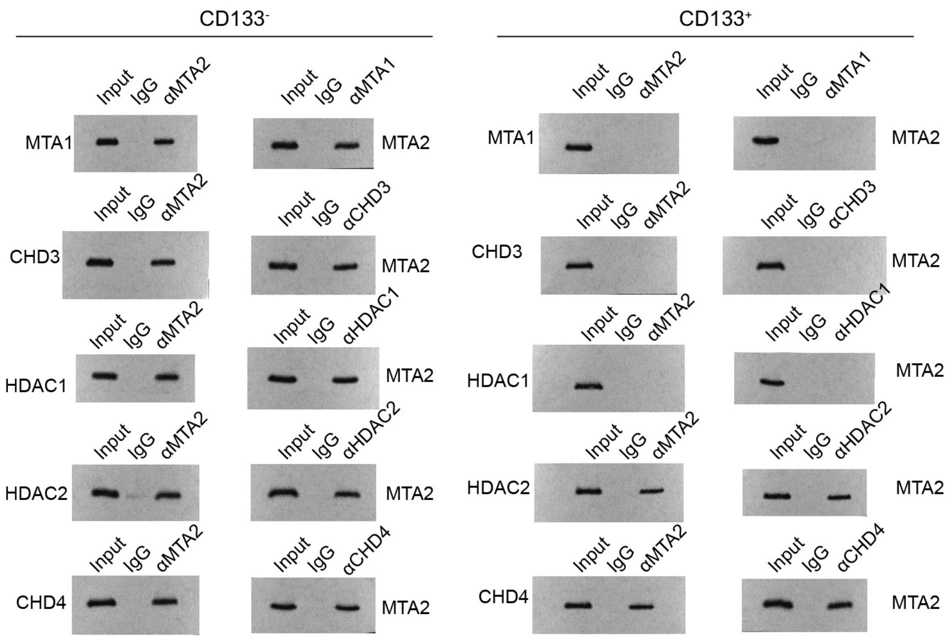

Fig. S12 Uncropped immunoblot image in Fig. S3D and Fig. S3E

REFERENCES

1. Meng, J. *et al.* Twist1 Regulates Vimentin through Cul2 Circular RNA to Promote EMT in Hepatocellular Carcinoma. *Cancer Res* **78**, 4150-4162 (2018).

2. Hamperl, S., Bocek, M.J., Saldivar, J.C., Swigut, T. & Cimprich, K.A. Transcription-Replication Conflict Orientation Modulates R-Loop Levels and Activates Distinct DNA Damage Responses. *Cell* **170**, 774-786 e719 (2017).

3. Sollier, J. *et al.* Transcription-Coupled Nucleotide Excision Repair Factors Promote R-Loop-Induced Genome Instability. *Molecular Cell* **56**, 777-785 (2014).

4. Hu, Y. & Smyth, G.K. ELDA: extreme limiting dilution analysis for comparing depleted and enriched populations in stem cell and other assays. *J Immunol Methods* **347**, 70-78 (2009).

5. Colaprico, A. *et al.* TCGAbiolinks: an R/Bioconductor package for integrative analysis of TCGA data. *Nucleic Acids Res* **44**, e71 (2016).

6. Yu, G., Wang, L.G., Han, Y. & He, Q.Y. clusterProfiler: an R package for comparing biological themes among gene clusters. *OMICS* **16**, 284-287 (2012).

7. Covington, K.R. & Fuqua, S.A. Role of MTA2 in human cancer. *Cancer Metastasis Rev* **33**, 921-928 (2014).

8. Shi, W. *et al.* Expression of MTA2 and Ki-67 in hepatocellular carcinoma and their correlation with prognosis. *Int J Clin Exp Pathol* **8**, 13083-13089 (2015).

9. Guan, C., Chang, Z., Gu, X. & Liu, R. MTA2 promotes HCC progression through repressing FRMD6, a key upstream component of hippo signaling pathway. *Biochem Biophys Res Commun* **515**, 112-118 (2019).

10. Fu, J. *et al.* The TWIST/Mi2/NuRD protein complex and its essential role in cancer metastasis. *Cell Res* **21**, 275-289 (2011).

11. Morey, L. *et al.* MBD3, a component of the NuRD complex, facilitates chromatin alteration and deposition of epigenetic marks. *Mol Cell Biol* **28**, 5912-5923 (2008).

12. Zhu, S. *et al.* Reciprocal loop of hypoxia-inducible factor-1alpha (HIF-1alpha) and metastasis-associated protein 2 (MTA2) contributes to the progression of pancreatic carcinoma by suppressing E-cadherin transcription. *J Pathol* **245**, 349-360 (2018).

13. Grunseich, C. *et al.* Senataxin Mutation Reveals How R-Loops Promote Transcription by Blocking DNA Methylation at Gene Promoters. *Mol Cell* **69**, 426-437 e427 (2018).

14. Garcia-Muse, T. & Aguilera, A. R Loops: From Physiological to Pathological Roles. *Cell* **179**, 604-618 (2019).

15. Crossley, M.P., Bocek, M. & Cimprich, K.A. R-Loops as Cellular Regulators and Genomic Threats. *Mol Cell* **73**, 398-411 (2019).

16. Wells, J.P., White, J. & Stirling, P.C. R Loops and Their Composite Cancer Connections. *Trends Cancer* **5**, 619-631 (2019).

17. Cristini, A., Groh, M., Kristiansen, M.S. & Gromak, N. RNA/DNA Hybrid Interactome Identifies DXH9 as a Molecular Player in Transcriptional Termination and R-Loop-Associated DNA Damage. *Cell Rep* **23**, 1891-1905 (2018).

18. Hanahan, D. & Weinberg, R.A. Hallmarks of cancer: the next generation. *Cell* **144**, 646-674 (2011).

19. Satriano, L., Lewinska, M., Rodrigues, P.M., Banales, J.M. & Andersen, J.B. Metabolic rearrangements in primary liver cancers: cause and consequences. *Nat Rev Gastroenterol Hepatol* **16**, 748-766 (2019).

20. Gingold, J.A., Zhu, D., Lee, D.F., Kaseb, A. & Chen, J. Genomic Profiling and Metabolic Homeostasis in Primary Liver Cancers. *Trends Mol Med* **24**, 395-411 (2018).

21. Puchalska, P. & Crawford, P.A. Multi-dimensional Roles of Ketone Bodies in Fuel Metabolism, Signaling, and Therapeutics. *Cell Metab* **25**, 262-284 (2017).

22. Cotter, D.G. *et al.* Ketogenesis prevents diet-induced fatty liver injury and hyperglycemia. *J Clin Invest* **124**, 5175-5190 (2014).

23. Martinez-Outschoorn, U.E. *et al.* Ketones and lactate increase cancer cell "stemness," driving recurrence, metastasis and poor clinical outcome in breast cancer: achieving personalized medicine via Metabolo-Genomics. *Cell Cycle* **10**, 1271-1286 (2011).

24. Martinez-Outschoorn, U.E. *et al.* Ketone body utilization drives tumor growth and metastasis. *Cell Cycle* **11**, 3964-3971 (2012).

25. Huang *et al.* Hepatocellular carcinoma redirects to ketolysis for progression under nutrition deprivation stress. *Cell Res* **26**, 1112-1130 (2016).

26. Young, M.E. *et al.* Cardiomyocyte-specific BMAL1 plays critical roles in metabolism, signaling, and maintenance of contractile function of the heart. *J Biol Rhythms* **29**, 257-276 (2014).

27. Major, J.L., Dewan, A., Salih, M., Leddy, J.J. & Tuana, B.S. E2F6 Impairs Glycolysis and Activates BDH1 Expression Prior to Dilated Cardiomyopathy. *PLoS One* **12**, e0170066 (2017).

28. Sabari, B.R., Zhang, D., Allis, C.D. & Zhao, Y. Metabolic regulation of gene expression through histone acylations. *Nat Rev Mol Cell Biol* **18**, 90-101 (2017).

29. Xie, Z. *et al.* Metabolic Regulation of Gene Expression by Histone Lysine beta-Hydroxybutyrylation. *Mol Cell* **62**, 194-206 (2016).

30. Apostolou, E. & Hochedlinger, K. Chromatin dynamics during cellular reprogramming. *Nature* **502**, 462-471 (2013).
